# Concentration as a Principle of Success or, "One Thing I Do."

**Published:** 1892-04

**Authors:** N. T. Roberts

**Affiliations:** VA.


AMERICAN JOURNAL

—OF—

DENTAL SCIENCE.

VOL. XXV. BALTIMORE, APRIL, 1892. No. 12.

ARTICLE I.—ORIGINAL.

### CONCENTRATION AS A PRINCIPLE OF SUCCESS OR, "ONE THING I DO."

AN ADDRESS TO THE GRADUATING CLASS BY REV. N. T. ROBERTS, OF VA.

*Gentlemen of the Graduating Class:*

I have been kindly invited to address you on this occasion, and I will say at once that I most cordially appreciate the honor of the invitation. I will also say at once that it has afforded me much pleasure, and pleasure of a very high order, to be with you to-day and to witness the interesting exercises which work the close of your College year. I do not doubt that the occasion has afforded just as much pleasure, and pleasure of just as high an order, to every person present as it has to your speaker. The bare presence of the large and distinguished audience which greets us this evening attests the *general interest* of the occasion, while the *general pleasure* which it affords is distinctly registergd upon the hundreds of beaming faces before me.

But, Gentlemen of the Graduating Class, to none present, I believe, whether it be your honored Dean, who, with keenest interest, has watched your course and with supreme satisfaction has marked your ready response to his daily instruction; whether it be some fond mother looking out from this audience with radiant face and tear-dimmed eye as she beheld the manly form of her boy come forward and receive the reward of honest work; whether it be a doting father, who feels to-day that he has a son of whom he has a right to be proud and to whom he can afford to be liberal, even to the extent of denying himself the actual comforts of life, in order that he may have the educational advantages which his studious habits and mental gifts so richly deserve; whether it be that most unselfish and lovable being upon earth, that born and blessing to any young man, that angel in human form, a sweet sister, came this evening to witness her big brother's first triumph in real life; or whether, last but not least, it be that adored and adoring one (I am not surprised to see you "catch on" to the allusion at once) whose image is enshrined in the inmost recesses of your hearts, who has been your inspiration in the long weary hours of severe application and tedious mastery of the various details of your profession and who is to be the inspiration and guiding star of whole future life, pleasure as the occasion undoubtedly is to each of these several classes who are all no doubt represented in this audience, yet to none of them is the occasion one of such *unique pleasure* as it is to you yourselves. *You feel a peculiar pleasure at this moment, because you know that you are giving pleasure to others* just at this moment. You are giving pleasure to your faithful and honored Faculty; you are giving pleasure to your fond, anxious mother; you are giving pleasure to your practical old father; you are giving pleasure to your sweet, unselfish sister; you are giving pleasure to your blushing, beautiful sweet-heart; you are giving pleasure, in fact, to every body. Therefore, you yourselves feel a *peculiar* pleasure. But in this present pleasure of yours—so full, so rich—there is a *new* and *strange* element. You are distinctly conscious of its presence, some of you—are you not? Other, perhaps, experience this *new* sensation of pleasure but feebly and uncertainly. Those of you who are distinctly conscious of a new element of pleasure in the experience of the evening hardly know, I suspect, what it means. You hardly know how to define it. Yon do not know what to call it. It is a distinctly *new* sensation. What is it? Permit me to tell you. It is the *thrill of victory*. It is the *throb of triumph* in your heart—the throb of a *first* triumph. You have met the enemy and they are yours. Features by the hundred and examinations by the dozen stood in the way of your graduation. Formidable obstacles they were—formidable enough to try your best metal. You went at them bravely. You surmounted them. You met the enemy and they are yours. And, gentlemen, the *consciousness of victory*, after a hard fought battle, makes your hearts beat quicker to-day, and makes the blood in your veins run faster, and sends a thrill and tremor of happiness through every fibre of your frame, so that you scarcely know your own selves, unless some practical joker nearby happens to remind you who you are by the careless way in which he handles a *pin*. Yes, you feel to-day something of that tumultuous whirl of pleasurable excitement, something of the thrill and throb of triumph, which the true soldier feels at the sight of the flying foe. He shouts for joy and tosses his old military cap high in air with one hand in utter forgetfulness for the moment that his other hand hangs helpless at his side, a bleeding mangled mass, the prey of the remorseless shell or the cruel minnie. And,if like Wolf at Quebec, one of you should now, in the very moment victory, with glowing face and throbbing breast, receive a summons which might not be postponed nor disobeyed,you might with perfect truthfulness adopt the little girl's version to the British General's last words and say, "*I never died so happy!*"

I trust, Gentlemen, that you may experience this *triumph-thrill* often in the life-struggle which is just opening up before you on such pleasant lines and in so glorious an era. May it come to you, over and over again, in your daily battle with the opposing forces of our physical and spiritual environments. And may the diplomas which you have so worthily won, and as gracefully received, be but the type and shadow, the prophecy and the pledge of what you will receive at the last great final of Earth, when all the graduates of the School of Christ, of every tribute and kindred and tongue, shall, before the assembled universe of angels and men, receive the public acknowledgment of meritorious work, and shall thenceforth forever wear the victor's crown and shall thenceforward forevermore enjoy the rest from toil and strife, which is the happy portion of all who are conquerors of sin and self.

But, Gentlemen, just here let me say that I trust you will pardon the representative and servant of the Church, one whose duty and privilege it is—whose *life* in fact it is—to minister to men in *holy* things; one whose duty it is to seek ever to turn the thoughts of men away from the earthly towards the heavenly, away from the material towards the spiritual, away from the seen towards the unseen, away from the transient towards the eternal; one whose duty and privilege it is to seek ever to make *all* the events and experiences in men's lives, both small and great, but especially the greater events and the larger and richer experiences of life, contribute to their spiritual enrichment—such an one, I trust, is excusable, aye, is justifiable in your eyes in taking advantage of the present moment and the present unique experience in your lives to introduce a brief time of somewhat serious but, it may be, allow me to hope, somewhat helpful thought.

I have dwelt at some length upon the pleasures of the evening. They have neither been invented nor overdrawn. They are very real. They are manifest and multitudinous. The public commendation of your able and honored corps of Professors; the congratulations of your friends and relatives; the applause of the general public; and above all, the consciousness on your parts of victory achieved by the means of manly, honorable toil— all of these things go to make up a day entirely unique, a day hitherto unknown in your pleasurable experiences. But if the day is unique in the *pleasure* which it affords, let me say, it is unique also in the *responsibility*, which it brings. *You have achieved a grand success.* This day is a witness of the fact. Will you *continue* to do as you have proved you can do? Will you *continue* to be successful? *You have made a good start.* The exercises of this evening publicly attest the fact. Will you *hold out* to the end of the race. Will you reach the goal that lies away out before you? Will you attain professional eminence and, by your distinguished services to suffering humanity, give more dignity and a brighter lustre to your already noble profession? I trust so, indeed. But do you know, gentlemen, that there is *danger* lurking in the *first* success of a young man? Many a young man has been ruined by a brilliant success at the start. And the more brilliant the first success the greater the danger that lurks in it. At this very moment there comes into mind the image of a man whom I knew in my college days, and whose brilliant gifts I fairly coveted. In a somewhat large and prolonged college experience he was by far the most brilliant man I ever met. A distinguished graduate of Princeton, he came to the University of Virginia to study law, and was easily first among four or five hundred fellow-students, both as an orator and as a writer. Apparently without effort he achieved the distinction of winning both the "Debator's Medal" and the "Magazine Medal" the same session. Of lofty presence and regal bearing, there really seemed no position of honor or trust in the gift of his countrymen which would not have been at once both honored and adorned by his presence. All who knew him confidently expected great things of this brilliant college graduate.

Alas, alas, they were doomed to disappointment. Years passed by, and I never heard or saw the slightest notice of the young man who had achieved such a brilliant success at college—who had made such a promising start in life. Last summer a distinguished Texas lawyer came to the valley of Virginia, and sought in repose and in the pure, fresh air and health-giving waters of our mountain town, relaxation of mind and invigoration of body. I was reminded of the brilliant collegian of former days, and was led to ask the San Antonia lawyer if he knew Jones. "Very well," he replied. I then mentioned Jones' college career, and remarked that I had expected to hear long before of his being in one of the halls of our National Capitol, using his rare gifts as a patriot in behalf of his country, and as a man in the interests of his kind. "Well," said the lawyer, in an expressive phrase which some of you may possibly have heard before, "*he will never get there.*" The fact of the business was that Jones' early success at college absolutely *ruined* him. After a brilliant dash, in which he distanced all competitors, he stopped short and rested on his oars. As a matter of course, slower but surer men soon overtook him and in the race of life he has been left far behind. He will probably never be heard of in any of the honorable or useful relations of life. So far he is a dead failure and likely to continue so.

Not so, I trust, with any of those whom I have the honor to address this evening. Never, never, never, I think, I hear you say to yourselves. Never, God helping us. Well said. I have no doubt you mean it from the bottom of your hearts. But, will you permit me, just at this point, to present for your consideration *one short fundamental principle of success*, enunciated by a name pre-eminent to-day throughout the civilized world, a man whose reputation has withstood the wear and tear of 1800 years, and whose form grows brighter with each revolution of the wheel of time. *Such* a name ought surely to have great weight with every rational thinking being. The principles of action which such a man lays down for his own guidance are surely worthy of the careful study of ordinary mortals like ourselves. You are perhaps familiar with Thomas Jefferson's *maxims.* There are ten of them. They constituted Jefferson's Decalogue of Practical Rules, and no doubt partly reveal the secret of the eminence which he attained in life, as well as the influence which he still exerts though long since mouldered into dust. They are full of practical wisdom and sagacity, and therefore I venture, both for the sake of contrast with a great *inspired maxim,* which I shall presently state, as well as for the sake of its own intrinsic value, to report the Sage of Monticello's Decalogue. First of all, he urges:

1. "Never put off till tomorrow what can be done to-day."

2. "Never trouble others to do what you can do yourself."

3. "Never spend your money before you have it."

4. "Never buy what you do not want because it is cheap."

5. "Pride costs as much as hunger, thirst and cold."

6. "We never repent of eating too little."

7. "Nothing is troublesome that we do willingly."

8. "How much pain those evils cost us which never happen."

9. "Take things by their smooth handle."

10. "When angry always count ten before you speak."

These rules, all must admit, are pre-eminently practical, and their daily application in real life would undoubtedly, I think, insure moderate success, if not eminence and enduring fame. But they are too numerous to be easily remembered. Time is short and you yourselves, no doubt, are eager to go forth from this building with some *one single axiomatic thought,* containing within the compass of a few words, the concentrated wisdom of practical experience and supernatural revelation. I shall, therefore, proceed at once to give you the controling principle of a life far grander, resulting in a form far more wide-spread and enduring than that even of America's confessed political thinkers. The man to whom I refer is St. Paul, the Christian Apostle and Martyr. In ever memoriable words, in a choice autobiographical passage in one of his Epistles, he says: "*One thing I do.*" These are memorable words, young gentlemen, and they reveal to us the scant power of a life which for fidelity and conviction, for passionate earnestness in a great and glorious cause, and for lasting beneficient results, has scarcely a parallel in all history.

"*One thing I do,*" says this man of power. What does he mean? He means to say that he did not dissipate and scatter and waste his energies. He has bonded and concentrated them on one object. He devoted himself to *one definite work*—the work of his *Apostleship*, the work to which he was called of God, his sacred profession, his holy vocation. To this work he devoted himself. From this definite work nothing whatever turns him aside. No opposition of man or devil, no privation, no persecution made him waver for one moment. "*One thing I do,*" was the axiomatic maxim of his life. He kept this maxim before him and he lived up to it. He became in consequence a man of extraordinary power. You want to learn the secret of that power. You want to acquire it and be able to exert it. As a help in this direction, let us then attempt to define a man like St. Paul—a man who, like the blessed Apostle, is able consistently to say, "*One thing I do.*" I do not think we shall be far wrong if we define a man, who makes these words of St. Paul his rule of conduct, as a man of *concentration.* I do not think we shall be far astray if we define St. Paul as a man of concentration. Hs was a man of *concentration,* and therefore he was a man of *power.* He must needs be the one before he could be the other. It is just so to-day. The man of concentration is to-day the man of power. The professional man who has the power to concentrate himself upon his profession is the man who is going to take the lead in his profession and who is going to hold it. This is the man who is going to reach the goal. He is certainly going to "get there," and no mistake. He is not going to be the one who "gets left." Not he. And just here I would venture to say that if any member of this present graduating class of sixty-three members will take the pains to analyze and ascertain the causes and conditions which most contributed to his being a member of this graduating class, rather than of next years, he will find that the most potent factor in the process, which made graduation possible, was *concentration.* You concentrated and you conquered. You concentrated your *time*, you concentrated your *thought*, you concentrated your *energies* on *one* thing. Unconsciously, you put into operation—successful operation too—the great principle which underlies the life of the greatest name in the history of Christianity—the name of its Divine Author alone excepted. St. Paul's *one thing I do principle* was adopted by you for the time, and we witness the happy result this evening. You concentrated, you conquered.

May I then, Gentlemen, ask you, at the stage of my remarks, to *recognize, once for all, the supreme value of concentrated effort?* The life of the blessed Apostle, St. Paul, illustrates its value, does it not? Your own recent experience, as it seems to me, confirms and emphasizes it. Conscientiously and distinctly, then, let me ask you, keep before you, by day and by night, when you rise up and when you lie down, when you work and when you rest, the absolute necessity, in order to the attainment of anything above the most common-place mediocrity in your profession, of concentrating your whole undivided energy upon your chosen, your peculiar, your own life work. To use the Apostle's phrase, let me urge you to recognize, now and forever hereafter, the incomparable value to you, as a principle and factor in your professional life, of the "*doing of one thing.*" Say to yourselves, over and over again,

"*One thing I do,*" until the thought becomes a normal, mental state, a fixed mental mode. When you have done this, you will have mustered the first principle underlying success in your chosen profession, you will have gone very far towards learning the secret both of power and of fame.

"*One thing I do.*" You begin to reoognize the value of the principle which the words embody. You begin to feel that you would like to be able always to put the principle into practice. Just here, then, I will drop one word of caution. Do not imagine that the habit of concentration and the power which is born of that habit is an easy thing to acquire. It is invaluable after it is acquired—the habit of concentration. Its acquisition means almost anything one wishes in the line of success; but the art of acquiring, the acquisition itself, will cost you both effort and self-denial. You have had some practical experience of this, no doubt, during the session just past. During the brief period you concentrated and you succeeded. But you had to make, daily, the positive distinct effort to concentrate. And then you had, daily, to deny yourselves a good many pleasurable things in order to carry out your daily mental resolve to concentrate yourself on your work. You had perhaps to cut down your visiting list somewhat, and it is quite possible that you found it necessary to deny yourselves many pleasant evenings out with "the boys." Is it not so? But you do not regret now. And, I think that I prophesy but truly when I say that, years from to-day when from the lofty height of professional fame you are able to look back upon a life in which you almost daily denied yourselves many of the petty passing pleasures which distract men, for the sake of concentrating yourselves, like the great Christian Apostle, wholly upon the vocation to which God in his providence called you, these self-denials will not cause you a moment's regret.

"*One thing I do.*" Do you catch the tone fully. Do you get a firm hold on the principle. I trust so. It is perfectly simple. At the same time it is fundamental and vital. "*One thing I do.*" I can give no wiser word, no sager advice than this maxim of St. Paul. It is the embodiment not only of the profoundest human sagacity, but of the highest heavenly wisdom. "*One thing I do.*" You have already experienced the value of these words, as an axiomatic principle underlying all success, in your first great triumph. Continue to apply the principle, and you will continue to triumph.

"*One thing I do.*" With these words I must now bid you go forth, no longer boys, no longer novices, no longer apprentices, but full fledged professional men, signed and sealed with the official sign and seal of your noble Alma Mater, fit and prepared in her judgment to take your places in the ranks of those who, under the seal of her approval, have already gone forth and are fighting, one here and one there, in dead earnest, the battle of real life—I say with these words, "*One thing I do,*" ringing in your ears and, I trust, indelibly impressed upon your minds, I bid you go forth. Go forth, Gentlemen of the Graduating Class, as consecrated men, consecrated to a mission of mercy to suffering humanity. Nothing less than this is your true mission. And what a high and holy mission is this to which you are called! There is no higher or holier mission on earth than a mission of relief to the suffering. To lessen the aggregate of human pain, to improve the aggregate of human health, to add to the aggregate of human beauty—this is your real, your high mission in life. Money-making merely is not your mission. Don't make this mistake. Don't degenerate into a mean money-maker. If you do, you will degrade your profession and rob your life of all nobleness of purpose. Don't lose sight of your real mission for one moment. Routine work will often tempt you to forget. Routine work must be done—some in every profession—routine work, often of the most common-place kind, often apparently of the most trifling character, often unattractive enough, this routine work must be done, it is a necessary evil of every profession, and it will often have a tendency to make you forget the nobility of your real, your true mission. Call to mind, then, often the real purpose to be subserved by the routine work of the office or laboratory, and work on under the inspiration of a noble purpose—a holy mission. Throw your whole soul into work. Be consecrated to it. Yes, as *consecrated men*, Gentlemen of the Graduating Class, I bid you go forth to your life work, and may God speed you in that work. Go and take with you, as you go, in addition to the inspired word which I have given you, take with you the memories of this happy hour. They will cheer you in your despondent moments, and refresh you in your weary ones. Such hours will probably come to most of you, as they have to all who have preceded you, and when they do come, then let memory take you back to this happy moment. Recall the smiling faces in this vast throng who have gathered here to witness your first triumph; recall the sweet strains of music which now sooth your spirit, now inspire you to action; listen afresh to the benediction of your Alma Mater; think once more of the grand life whose fundamental axiom I have so briefly and so imperfectly unfolded; groop afresh the inspired word of the Christian Apostle and Martyr, "*One thing I do.*" Do this and you will probably again feel the thrill and throb of your first triumph, and you will go forth again conquering and to conquer.

God bless you, young men, and make the light of His countenance to shine upon and give you peace.

